# The evaluation of surface sealants’ effect on the color stability of Nano-hybrid composite after polishing with One-Step system (in-vitro)

**DOI:** 10.4317/jced.54857

**Published:** 2018-09-01

**Authors:** Kaveh Khalaj, Armin Soudi, Mahsima Tayefi-Nasrabadi, Mohammad-Ali Keshvad

**Affiliations:** 1 Assistant Professor of Operative Dentistry, School of Dentistry, Tehran University of Medical Sciences, Tehran, Iran; 2 DDS, Department of Orthodontics, School of Dentistry, Tehran University of Medical Sciences, Tehran, Iran; 3 DDS, Department of Oral and Maxillofacial Radiology, School of Dentistry, Shahid Beheshti University of Medical Sciences

## Abstract

**Background:**

The clinical use of composite resins has increased substantially over the past few years due to increased esthetic demands by patients, improvements in formulation, and simplification of bonding procedures. An alternative for preventing or reducing composite discoloration is using of composite surface sealant. The purpose of this study was to determine the effect of surface sealant on color-stability of Nano-hybrid composites after polishing with one-step system.

**Material and Methods:**

56 disk-shaped specimens (10*2 mm) of a Nano-hybrid composite resin (VOCO,Cuxhaven,Germany) were prepared. The specimens were randomly divided in to 2 groups of 28 specimens each. The surfaces of specimens were polished with One-step system (Dimanto,VOCO,Germany ). one group of specimens assigned as control group and received no more surface treatment .surface sealant (PermaSeal,Ultradent,USA) was applied on the surface of specimens of the other group as experimental group .specimens were stored in Ringer’s solution for 24 hours at 37oC. The specimens were subjected to artificial aging with thermocycling method between coffee solution at 55°C and Ringer’s solution at 5°C for 3000 cycles. CIE L*a*b color coordinates were established using a spectrophotometer for each specimen. Statistical significance was set at *p*<0.05.

**Results:**

After aging in both groups, color changing was significantly different (*p*<0.05) and clinically unacceptable (ΔE> 3.3) and there was no significant difference between the two groups in ΔE.

**Conclusions:**

Surface sealant was not effective in improving color stability.

** Key words:**Surface-penetrating Sealant, Color Stability, Nano-hybrid composites.

## Introduction

The Acid-etch technique was introduced by Buonocore in 1955, later in 1956, Bowen introduced resin Composites (1,2). The development of resin composites has led to the inclusion of aesthetic aspects of dental direct restorations and met the demands of patients who are not only expecting a dentist to reconstruct their teeth with their restorative materials, but also require restorations that are tooth-colored and not detectable (3,4).

When the dentist wishes to achieve a direct restorations consistent with the dental structure, the resin composite is the preferred restorative material (5). However, due to its inability to withstand abrasion, it does not give the desired results to consider as an ideal material (6). Resin composites show some undesirable properties such as microleakage due to polymerization contraction (leading to marginal leakage) (3,6). In addition, the composites undergo surface and intrinsic changes, which are influenced by the chewing forces and finishing processes (3). The advantages of composites, in addition to its esthetic, include corrosion resistance, no electrical conductivity and mercury-free structure (5,7).

Resin Composite restorations are affected by color change due to the aging process. The studies point out that this effect may be related to factors such as the formulation of the substance, finishing processes, and the coloring factors involved in the restoration (8). Resin composites are prone to color changes in in-vivo and in-vitro environments when exposed to common colored materials such as beverages, coffee and tea (9). Color stability is critical to success in any esthetic restoration, in fact color change is the main reason behind the replacement of anterior restorations (10). Therefore, in order to increase the durability of resin composites and avoid the need for their replacement due to color variations, experts suggest possible solutions to remove or reduce their coloration. Surface sealants are possible solution for removing or minimizing the color variations of resin composites, which include BIS-GMA, UDMA and TEG-DMA without filler particles. It has a low viscosity and high wetting ability (11,12). Sealants are used to fill the surface roughness on the surface of composite restorations to improve surface luster, marginal seal and abrasion resistance. The use of surface sealants may also affect the absorption of pigments and the color stability of composite restorations (13).

In the definition and reception of the color three words Hue, Chroma and Value are used. Value is the biggest factor in color determination. It is easier to determine the differences of value. Because the number of cylindrical cells in the eye is more than conic cells (14).

CIE System: Apart from the Mansell, CIE numerical systems is also used to determine color. The CIE system is the most commonly used system and is based on the Commission internationale de I’Eclairage. CIE presented its first color system in 1931. Although the system has been modified over the years, its general principles remain unchanged. The basis of CIE’s work is to determine the numerical value of three color stimuli. The creation of color has three factors: the source of light, the object and the observer (15). The color defined using the CIE system can be defined on the color diagram X versus Y, as shown below. This diagram, which is like a horseshoe, is the geometric location of all colored points, and is referred to as Lucas Spectrum.

In this system, Y represents the brightness, it is positioned as a perpendicular axis. The line connecting the dots represents the color spectral axes. In order to communicate more with visual estimates, since 1976, the Lab color system was used instead. In this system: “b” represents blue-yellow (b +: yellow, b-: blue), “a” green-red (a +: red, a-: green) “L” represents the brightness. Lab values can be obtained using the following relationships from x, y, z. (16)

L*=116f(Y/Yn)-16

a*=500[f(X/Xn)-f(Y/Yn)]

b*=200[f(Y/Yn0-f(Z/Zn)]

**Figure 1:**
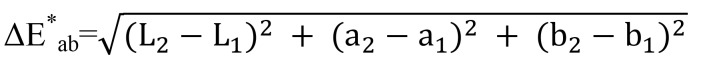
Formula.

The color difference between the two samples in this system is determined using the ΔE value from the following equation, (Fig. 1).

Color stability of dental restorative materials depends on factors such as degree of conversion, water absorption, chemical reactivity, diet, oral hygiene, and surface roughness. Staining may result from a chemical reaction between the surface of the resin and the colored materials, the surface of the resin composite, which is polymerized weakly, shows a higher surface reaction. Newly polymerized surfaces are more susceptible to color than older ones (17). The nanomers is a 25 to 75 nm invisible particles, and the nanoclusters include silica-zirconia cross-linked nanoparticles, which result in an average 0.6 μm in size. By combining nanoparticles and nanoclusters, this formula reduces in-network spaces in inorganic particles, and provides better physical properties and reduces the disintegration of the material over the years. The technology also achieves mechanical properties that allow it to be used both in the anterior teeth and posterior teeth. Most researchers have proven that nanofilled and Nano-hybrid composites have high color stability and can keep their surface luminosity at a high level. Although color stability in the oral environment under the influence of strong dye fluids remains questionable (18-20). Therefore, it is important to determine whether the use of surface sealant of Nano-hybrid composites offers better results for esthetic restorations or not. The purpose of this study was to determine the effect of surface sealant on the color stability of Nano-hybrid composites after a one-step polishing system under aging processes in in-vitro environment.

## Material and Methods

A total of 56 composite (Grandio,Voco, Cuxhaven, Germany) with A2 color, were made using medical grade silicon mold. To make these samples, the composite was deposited in a layer inside the mold using the Optra Sculp (Ivoclar-vivadent) instrument. To ensure a smooth surface in the samples, before light curing, the upper surface of the mold was covered with a transparent matrix and another glass slab was placed on it, and to remove the composite additions and to create a smooth standard surface, one- Kilogram weight for 30 seconds was placed on the set. After that time, material was cured by a Woodpecker light cure device with the intensity of 800 mw / cm2 for 40 seconds according to factory order. The tip of the device was placed at a distance of less than 5 mm perpendicular to the composite surface, and the intensity of the output light of the device was initially monitored by the radiometer device and among each 5 samples.

Then, the surface of the samples were polished by lens disk of one-stage Dimanto polishing system (VOCO), with a low-speed handpiece, without water spray for 20 seconds according to factory order. Initially, the average pressure was applied, and in the final stage of polishing, the resulting pressure dropped to achieve the gloss. Polishing all the samples to avoid interpersonal differences was done by a trained person. Then, the samples were thoroughly washed and dried.

The samples were randomly divided into two groups of 28, a group of samples was selected at this stage as a control group, and on the other group (experimental group) etching was performed for 20 seconds by phosphoric acid 37% (VOCO, Germany), then washed with water for 20 seconds, and dried with absorb paper and air pressure. A thin layer of surface sealant PermaSeal (Ultradent, USA) was placed on the surface of the sample using a Black Micro FX (Ultradent, USA) tip, and was rubbed onto the composite surface with rubbing motions for 5 seconds, with gentle pressure of air was thinned and cured for 20 seconds according to the order of the factory. Then, the samples were removed from the inside of the mold, and the lateral additions of the composite and surface sealant were slowly removed using a diamond 008 fissure bur.

All samples were kept in Ringer’s solution for 24 hours in a Kavoosh MEGA incubator (Kavoosh industry,Tehran,Iran) at 37 ± 1 ° C.

The X-Rite SP62 Portable Sphere Spectrophotometer (Michigan, USA) was used in a range of 400 to 700 nm. First, the spectrophotometer was calibrated in white and then in black. Then each of the samples was removed from the ringer solution, washed with water pressure for 5 seconds, and the moisture was dried using absorbent paper and placed in the spectrophotometer on a white background to remove the background light and Color measurements were made at the center of each composite disc. The values of L, a and b were written for each sample as the initial values of the composite color. The color measurement was repeated three times, and the average of the three obtained values was calculated. The spectrophotometer was re-calibrated in white and black in each of the five samples 

The TC-300 Thermo-cycle (Vafaei industrial, Iran) was used for aging the samples. A standard coffee solution was prepared by dissolving 20 grams of Nescafe in 250 ml of boiling water. Samples were subjected to intermittent thermal stresses in Thermo-cycle in 5 ° C water and 55 ° C coffee solution suspended at 3000 cycles (with a transfer time of 10 seconds between two baths and 30 seconds in each bath).The coffee solution was replaced after every 500 cycles. After completion of the thermo-cycle, the samples were washed with distilled water and dried with moisture absorbent paper.

To measure the final color of the samples, the spectrophotometer was calibrated according to the black and white method. The color of each sample was repeated 3 times, and the mean values were calculated. The values of L, a, b were written for each sample as the final color. The spectrophotometer was re-calibrated in white and black in each of the five samples. Data were analyzed by SPSS20 software. After descriptive statistics, t-test, chi-square test was used. P value less than 0.05 was considered as significant difference.

## Results

The *p* value (0.00) indicates a significant difference (this value is compared with 0.05, if the number is smaller than 0.05, then the difference is significant, otherwise two groups do not have significant difference) between the a* and b* color systems before and after the aging process in experimental group and this amount is greater after the aging process. *p* value (0.00) indicates that there is a significant difference between the color system l* before and after the aging process in experimental group and this amount is less after the aging process ( Table 1).

**Table 1:**
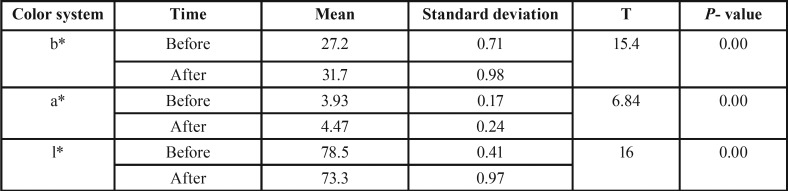
Comparison of the b*, a* and l* color system before and after the aging process in Experimental Group.

Using paired t-test, we obtained *p* value=0.00 which indicates a significant difference between the a* , b* color systems before and after the aging process in control group which is higher after the aging process. There is a significant difference (*P* =0.00) between the color system l* before and after the aging process in the control group, and this amount is less after the aging process ( Table 2).

**Table 2:**
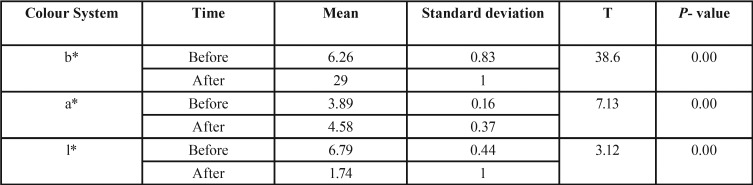
Comparison of the b*, a* and l* color system before and after the aging process in Control Group.

Using independent t test, The *p* value (0.001) indicates that there is a significant difference between b* and a* color systems in the one-step polishing with and without applying the sealant after the aging process, according to the mean values it can be concluded that this value in the control group is less than the experimental group. There is a significant difference (*p* value = 0.016) between a* in two groups during the aging process, according to the mean values it can be concluded that this value in the control group is more than the experimental group. there is no significant difference between the l* values (*p*-value=0.69) in two groups ( Table 3).

**Table 3:**
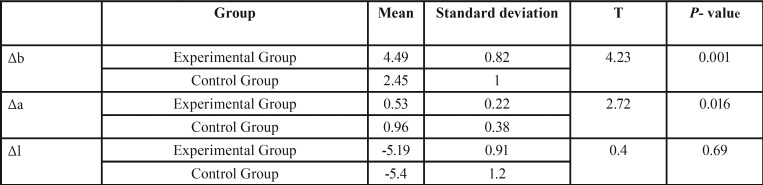
Comparison of Δb, Δa and Δl during the aging process between the Control and Experimental groups.

There is no significant difference between the color stability difference (ΔE) in the control and experimental groups (*p* value = 0.19).

## Discussion

Color stability is one of the important qualitative factors in composites. Changing color in the composite depends on the resin matrix, the percentage of the filler particles, the adsorption and absorption of the colors, the type of solution and the chemical reaction between the composite and the dyestuffs (2,19-21). The color change of resin materials has both internal causes and external causes. Color change due to aging is related to the internal color variation (22). Surface sealant is a possible solution for removing or minimizing the color variations of composite resins, which include BIS-GMA, UDMA and TEG-DMA without filler particles. This material has a low viscosity and high functionality (3,12). The surface sealant appears to fill the surface microstructural defects and reducing the surface roughness and bubble, There are different views on surface sealant, which improves surface properties, resistance to color change and the visual characteristics of composite resin (23-25). Recently, one-step polishing systems have been introduced, with the benefits of less chairside time, minimal surface roughness on Nano hybrid composites, and thus a reduction in the likelihood of coloration (13,26).

In our study, a coffee solution was used to check the color stability of the samples. This selection was based on the results of Pennazo *et al.*, Which showed that the coffee produced the highest coloration in the composite among the colored solutions studied (6).

Aging can cause structural changes in material composition and affect its performance. In this study, we tried to simulate the environmental conditions of the composite over time in the oral environment. Therefore, in addition to immersion of the samples in a colorant solution, simulation of thermal changes was also used. Our samples were subjected to 3000 cycles of thermo-cycling between a 55 ° C coffee solution and a 5 ° ringer solution (27).

Spectrophotometer was used with the CIE L * a * b method to measure ΔE in most studies (6,9,27,28). This electronic device, unlike the calorimeter, uses a standard light in a dark component away from the ambient light to measure color, and thus the results are certain, definite and repeatable. ΔE>3.3 is a clinically significant and unacceptable color change (2,8,10).

The results of this study showed that the aging process significantly increases the composite color. The *P* values obtained for L, a, b parameters indicate a significant statistical difference before and after the aging processes in both control and experimental groups (*p* <0.05), in a way that the values of a, b, and l had decreased. Δb was higher in the experimental group and Δa in the control group was higher. However, the difference in Δl in both groups was not significant (*P* = 0.69). Changes in the color of both groups were not clinically acceptable (Δ E≥3.3) and ΔE difference between two groups was not statistically significant (*P* = 0.19), but in the experimental group (with sealant), it was more than the control group. These results were consistent with the results of the Catelan *et al.*, Lee and Powers, zimmerli *et al.*, which determined the effect of surface sealant on the color stability of the composites (28-30).

Lee and Powers reported the use of surface sealants (BisCover) to determine the resin composite color stability. They stated that the effect of the sealant on the composite is more dependent on its surface properties than the sealant properties. On the other hand, the used monomer in this sealant was responsible for its colorfulness (29). The disadvantage of this study was the low immersion time (3 days), which, according to Garcia *et al.*, the shortest time to measure the color stability is one week (3). Zimmerli et al. studied the effect of coffee and brushing on surface sealants, and concluded that the composite samples containing this material and samples without this material did not have a significant difference in ΔE. They stated that the cause of this result is debond and degrading of the sealant from composite samples (30).

Onur Shahin *et al.* determined the effect of surface sealant on the color stability of artificial teeth, a group had sealant and the other group was prepared with usual methods, and then the samples were thermo-cycled. They concluded that surface sealant improves color stability, which was opposite of our results. The reason for this can be the difference in the type of tested materials, they used PMMA and reinforced-PMMA and used fewer cycles (1000 cycles) for thermo-cycling (31).

In order to explain the unacceptable color variation obtained in our study, we can mention the high temperature of the coffee solution (55°C), which probably affects the surface of the material and degradation of the organic matrix, and thus it has facilitated pigmentation. Also, this result can be due to the absorption of colorants into the organic phase due to the compatibility of the polymer phase with yellow colored coffee and the reduction of color stability due to higher levels of hydrophilic comonomers in the surface sealant (3). Probably the other reason is that, unlike resin composites, there are no filler particles in the surface sealant resin matrix; the filler particles are neutral and do not absorb the liquid or dye (8).

Another factor that may reduce the surface sealants’ effectiveness is the thickness of the composite. According to Lee *et al.* the higher thickness of applied surface sealant on the composite is more susceptible to color changes, due to its viscosity and wetting properties, it is not possible to apply the thickness of the sealant to the same standard as the whole surface of composite (31). This limitation also exists in clinical situations.

## Conclusions

It can be concluded that the surface sealant is not efficient to protect against the color change of the Nano-hybrid composite, and even the amount of color change rate is higher than control group.
